# Gemella morbillorum Cryptogenic Brain Abscess: A Case Report and Literature Review

**DOI:** 10.7759/cureus.3612

**Published:** 2018-11-19

**Authors:** Ahmad A Abu-Heija, Mustafa Ajam, Jennifer Veltman

**Affiliations:** 1 Internal Medicine, Detroit Medical Center - Wayne State University, Detroit, USA

**Keywords:** brain abscess, gemella morbillorum, cryptogenic brain abscess, atrial septal defect

## Abstract

A case of cryptogenic brain abscess caused by *Gemella morbillorum* is reported in a 28-year-old immunocompetent man who presented with seizures and hemiparesis. The patient underwent successful stereotactic drainage of the abscess with complete resolution of symptoms and radiographic evidence of resolution. We report the significant pathogenic potential of a normal commensal rarely identified in neurologic infections.

## Introduction

*Gemella morbillorum* is a facultatively anaerobic, non-motile and non-spore forming Gram-positive coccus. The bacterium was first isolated by Tunnicliff in 1917 [1]. *G morbillorum* was known as *Streptococcus morbillorum* for most of the 20th century, until 1988 when, based on the biomolecular features of the bacterium, it was transferred to the genus *Gemella* as *Gemella morbillorum*. *G morbillorum* is commonly found as part of the normal flora of the oropharynx, gastrointestinal tract and female genital tracts [2]. *G morbillorum* has been recognized as a possible pathogen in more recent years, with documented cases of endocarditis, septic shock, liver abscess, and meningitis, seen in the literature [3].

Brain abscesses are devastating neurological conditions that require a multi-disciplinary approach with high clinical suspicion to enable prompt and effective management. Brain abscesses arise from either contiguous spread or a hematogenous source, and many remain labeled as cryptogenic after extensive evaluation. An intra-cardiac shunt can allow bacteria to bypass pulmonary filtration, providing them access to the systemic circulation.

## Case presentation

In our case, we present a 28-year-old gentleman with a remote medical history of seizure disorder five years ago, who was transferred to our facility for further evaluation after presenting with tonic-clonic seizures and new-onset right-sided hemiparesis. The patient was afebrile, and did not report a history of recent infections, injuries or injection drug use. Neurological examination revealed an awake and oriented middle-aged male, with a paucity of speech, motor strength 1 of 5 in the right upper and lower extremities. Further examination of the oral cavity, oro- and naso-pharynx revealed poor dentition with no signs of localized infection.

**Figure 1:**
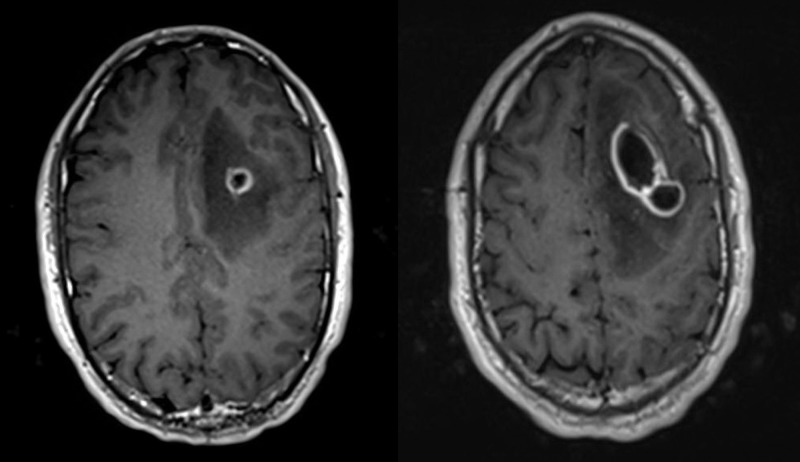
Brain magnetic resonance imaging (MRI) on admission. Brain MRI T1 Axial images showing focal restricted diffusion centered at the left frontal lobe at the grey-white matter junction with surrounding rim enhancement with significant perilesional vasogenic edema with mass effect on the frontal horn of the left lateral ventricle.

Biochemical and hematological investigations revealed a normal leukocyte count (4,500/µL, normal 3,500-10,600), normal C-reactive protein level (4.22 mg/L, normal <9.10) and normal erythrocyte sedimentation rate (8 mm/hr, normal 0-13). Computed tomography (CT) of the head showed three adjacent ring-enhancing lesions in the left frontal lobe with 5 mm midline shift to the right, brain magnetic resonance imaging (MRI) T1 Axial sections showed a multi-loculated enhancing lesion with restricted focal diffusion surrounded by perilesional vasogenic edema with mass effect on the frontal horn of the left lateral ventricle (Figure 1).

Intravenous (IV) corticosteroids were administered for their anti-inflammatory effect to hinder further edema, and empiric antibiotics were initiated with vancomycin, ceftriaxone, and metronidazole. Human immunodeficiency virus (HIV) infection was excluded. In an attempt to localize the primary source, CT of the sinuses did not reveal any evidence of sinusitis. CT of the abdomen and pelvis did not reveal any intra-abdominal abscesses. Trans-esophageal echocardiography revealed no evidence of valvular vegetations, however, it revealed a large sinus venosus atrial septal defect. CT angiography revealed contrast extravasation from the superior vena cava to the right superior pulmonary vein, suggestive of a right-to-left shunt.

The abscess was drained via a frontal craniotomy with stereotactic assistance; purulent fluid was evacuated and sent for culturing. Anaerobic cultures grew *G morbillorum* and *Peptostreptococcus* species. We used an initial antibiotic regimen consisting of a combination of ceftriaxone, metronidazole, and vancomycin. Antimicrobial susceptibility testing was not done, given the lack of standardized testing and no interpretation criteria. The patient started to develop fevers on post-operative day 7; the decision was made to discontinue vancomycin given the lack of methicillin resistant staphylococcus aureus (MRSA) growth in tissue cultures. Antibiotics were de-escalated to penicillin G and metronidazole, for a six-week course. Fever defervesced 24 hours after switching to penicillin, suggesting adequate sensitivity. Decision to use penicillin G was made after results for syphilis testing returned positive. On discharge, 17 days from presentation, the patient had a dramatic improvement in muscular strength, with bilateral motor function at baseline.

**Figure 2:**
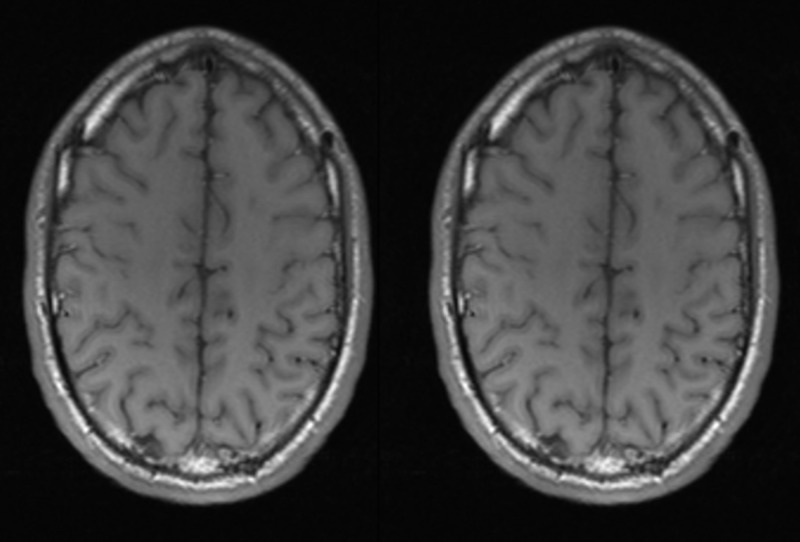
Brain magnetic resonance imaging (MRI) follow-up at four months. Brain MRI T1 Axial images showing resolution of the previously seen focal restriction and vasogenic edema.

Our patient was discharged home to complete a six-week course of penicillin G and metronidazole. Antibiotics were continued for a course of six weeks. The patient was followed up with further imaging to ensure complete neurological resolution of the brain abscess. Brain MRI was done four months after presentation showing near-complete resolution of the abscess with significant improvement of brain edema (Figure 2).

## Discussion

Microorganisms responsible for brain abscess formation are directly related to the primary source of infection. Oral flora, including *G morbillorum*, is mostly linked to an odontogenic disease process. Approximately 55% of the reported cases of brain abscesses caused by *G morbillorum* had an odontogenic primary source, 36% had an unknown primary source, and 9% (one case) was linked to septic arthritis [4,5]. Table 1 shows the reported cases in literature along with the reported source of infection, the location of abscess(es), presentation, antibiotics utilized and outcome.

**Table 1:** Reported cases of cerebral abscesses caused by Gemella morbillorum.

Reference	Case	Reported Source	Intracranial Location	Presentation	Empiric Antibiotics	De-escalated Antibiotics	Outcome
Asensi et al. [4], 1996	57 male	Periodontitis	Frontoparietotemporal	Fever, headache, hemiparesis	Imipenem	Imipenem	Cure
Murray et al. [5], 1998	45 male	Septic arthritis	Frontal	Acute meningitis	Ceftriaxone, Amoxicillin	Ceftriaxone, Metronidazole, Gentamicin	Cure
Messori et al. [6], 2002	28 male	Sinusitis dental extraction	Frontoparietal	Fever, headache, neck stiffness	Amoxicillin-Clavulanic acid, Clindamycin	Vancomycin, Chloramphenicol	Cure
Spagnoli et al. [7], 2003	47 male	Dental abscess	Frontoparietal	Fever, headache, hemiparesis	Ceftriaxone	Amoxicillin-Clavulanic acid	Cure
Spagnoli et al. [7], 2003	40 male	Unknown	Frontal	Fever, seizure, gait and speech disturbances	Ceftazidime	Amoxicillin-Clavulanic acid	Cure
Liberto et al. [8], 2006	75 female	Dental abscess	Frontal	Fever, headache, vomiting	Meropenem, Metronidazole	Meropenem, Metronidazole	Cure
Lopes et al. [9], 2007	50 male	Dental procedure	Cerebellar	Fever, headache, gait ataxia	Vancomycin, Ceftriaxone, Metronidazole	Metronidazole	Cure
Benedetti et al. [3], 2010	77 male	Unknown	Cerebellar, multiple	Fever, headache, gait ataxia	Multiple combinations	Multiple combinations	Death
Chotai et al. [10], 2010	67 male	Periodontitis	Frontal	Hemiparesis	Ceftriaxone, Metronidazole	Ceftriaxone, Metronidazole	Cure
Milnik et al. [11], 2013	39 male	Unknown	Parietal	Headache, numbness	Cefotiam, Metronidazole, Vancomycin	Cefotiam, Metronidazole	Cure
Our case 2017	28 male	Unknown	Frontal	Seizure, hemiparesis	Ceftriaxone, Vancomycin, Metronidazole	Penicillin G, Metronidazole	Cure

**Figure 3:**
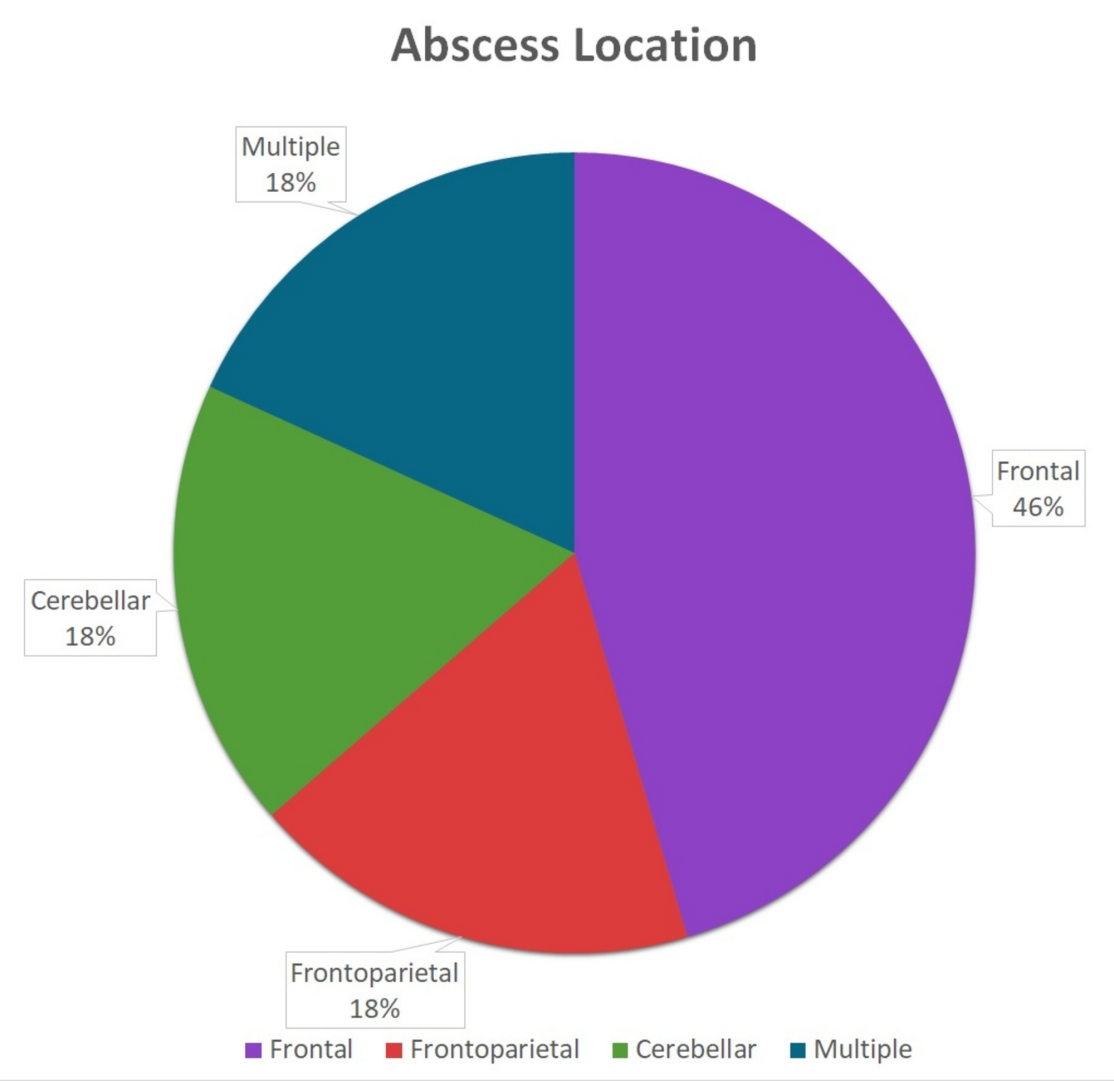
Anatomic location of abscesses reported in the literature.

Underlying conditions such as congenital heart defects, immunosuppression, poor dentition, as well as previous invasive medical procedures were associated with human infection with *G morbillorum*; however, the reports of multiple cases in previously healthy individuals reckon its pathogenicity of more burden than presumed [6]. Frontal lobe involvement was most commonly documented, with approximately 45% of the cases involving the frontal lobe, as seen in Figure 3.

In our case, the maxillofacial CT scan did not reveal any evidence of a sinus or a dental abscess as a likely source for the bacterium, thus a hematogenous spread was presumed the culprit of our infection with likely transient bacteremia caused by dental manipulation, or minimal dental trauma given his poor dentition [12]. As further investigations using CT angiography of the aorta and coronaries revealed contrast extravasation from the superior vena cava to the right superior pulmonary vein, suggestive of a right-to-left shunt, as well as a large sinus venosus atrial septal defect, revealed on trans-esophageal echocardiography, with evidence of intra-cardiac shunting. Right-to-left shunting as seen in various cardiovascular anomalies such as an atrial septal defect (ASD), patent foramen ovale (PFO), or anomalous pulmonary venous connection (partial or complete) may allow the passage of bacteria unhindered, avoiding the filtration mechanisms of the pulmonary circulation resulting in passage of a larger bacterial inoculum into the systemic circulation and the cerebral circulation resulting in abscess formation [13].

Infective brain abscesses are mostly seen in adolescents and children with congenital heart defects; adults with the aforementioned heart defects are also at risk [5]. Thus, we advise screening for congenital heart diseases whenever faced with a brain abscess with organisms usually seen as commensals of the oral flora, in the absence of apparent sinus or dental disease; they should also be advised to maintain oral hygiene to minimize oral bacterial load as simple manipulation or trauma can result in transient bacteremia with devastating results [12].

Presentation of patients with *G morbillorum* central nervous system (CNS) infections ranges along a spectrum of non-specific signs and symptoms including fever, headache, nausea, and vomiting, to more specific motor deficits, seizures, and meningeal signs. Majority of cases in the literature presented acutely, however, a more insidious pattern was also recognized, as reported by Benedetti et al. [3], with a biphasic clinical course, with an acute presentation with meningitis without a focal lesion seen on CT imaging, followed shortly by abscess formation in the cerebellar and cerebral parenchyma despite broad antimicrobial coverage. A similar pattern was reported by Murray et al. [5]. Suggesting latent CNS infection mimicking meningitis followed by suppuration and abscess formation in both cases, thus a prolonged course of antibiotics, six weeks, is advised in preference to a shorter duration of treatment.

Diagnostic imaging studies for brain abscesses classically included CT scan with contrast. However, MRI proved greater benefit in diagnosing brain abscesses given its ability to differentiate an abscess from other ring-enhancing lesions such as tumors. Furthermore, it can help predict the degree of associated inflammation. In addition, imaging enabled us to distinguish a cerebral abscess from a possible syphilitic gumma masquerading as an abscess. In our case, CT head with contrast revealed a focally restricting, distinctive ring-enhancing lesion without homogenous intralesional enhancement, as would be expected with syphilitic gummata. In addition, syphilitic gummata tend to develop adjacent to cerebral convexities, with meningeal encroachment, however, our patient lacked such characteristic attributes. Furthermore, evacuation of pus from the abscess via the frontal craniotomy, provided more evidence as to the etiology of the abscess.

Successful treatment of brain abscesses includes combined surgical and prolonged medical treatment (4-6 weeks). Both needle aspiration and surgical excision of the abscess have been used for therapeutic and diagnostic purposes. Early efforts for microbiologic diagnosis should be made for appropriate antimicrobial planning. Empiric treatment of brain abscesses most commonly included beta-lactams (e.g., penicillin, amoxicillin-clavulanic acid, ceftriaxone), metronidazole to cover anaerobes and vancomycin for MRSA coverage.

Majority of cases of *Gemella morbillorum* were cured with antibiotic regimens inclusive of a third-generation cephalosporin (e.g., ceftriaxone or cefotaxime) and metronidazole, with few reports adding vancomycin empirically. Use of metronidazole is questionable in such abscesses, but its broad-spectrum activity in addition to its low cost and relatively low risk of adverse events justifies its use in such cases [10].

## Conclusions

Our case presents a clinically challenging case with an immunocompetent young male developing sudden devastating neurological compromise, attributed to a frontal brain abscess caused by *Gemella morbillorum*. Our case was successfully treated with a total of six weeks of penicillin G and metronidazole. Timely diagnosis and management of the brain abscess are of the essence in order to limit the neurological sequalae caused by the infection. Adequate clinical follow-up with imaging is necessary to ensure the eradication of the organism and addressing the underlying source of infection should be emphasized to prevent recurrent infection and minimize morbidity.
